# Chinese culture permeation in the treatment of Parkinson disease: a cross-sectional study in four regions of China

**DOI:** 10.1186/1756-0500-7-65

**Published:** 2014-01-30

**Authors:** Zhen-Xin Zhang, Honglei Chen, Sheng-Di Chen, Ming Shao, Sheng-Gang Sun, Qiu-Min Qu, Bao-Rong Zhang, Yi-Ming Liu, Qun Xu, Xia Wan, Ling Li, Hong-Bo Wen, Xia Chen, Hai-Bo Chen, Zhen-Guo Liu, Jian Wang, Gang Wang

**Affiliations:** 1 Departments of Neurology, Peking Union Medical College Hospital, and Chinese Academy of Medical Sciences, 1 Shuaifuyuan, Wanfujing, Beijing 100730, China; 2 Beijing Brain Health Center, Beijing, China; 3 Epidemiology Branch, National Institute of Environmental Health Sciences, National Institutes of Health, Research Triangle Park, North Carolina, USA; 4 Department of Neurology, Ruijing Hospital, Shanghai Jiaotong University, Shanghai, China; 5 Department of Neurology, First Affiliated Hospital, Guangzhou Medical College, Guangzhou, China; 6 Department of Neurology, Xiehe Hospital, Wuhan, China; 7 Department of Neurology, First Affiliated Hospital of Medical College of Xi’an Jiaotong University, Xi’an, China; 8 Department of Neurology, Second Affiliated Hospital, Zhejiang University, School of Medicine, Hengzhou, China; 9 Department of Neurology, First Hospital, Shandong University, Jinan, China; 10 Department of Epidemiology and Biostatistics, Institute of Basic Medical Science, School of Basic Medicine, Peking Union Medical College, Chinese Academy of Medical Sciences, Beijing, China; 11 Department of Neurology, Beijing Hospital, Beijing, China; 12 Department of Neurology, Xinhua Hospital, Shanghai Jiaotong University, Shanghai, China; 13 Department of Neurology, Huashan Hospital, Shanghai Fudan University, Shanghai, China

**Keywords:** Parkinson disease, Treatment, Dyskinesias, Motor fluctuations, China

## Abstract

**Background:**

Little is known about the clinical features and treatment of Chinese patients with Parkinson disease (PD).

**Methods:**

A large cross-sectional survey of clinical features, medication use, and motor complications was conducted in 901 consecutive PD patients, from 42 randomly selected university-affiliated hospitals in four urban economic regions of China, between December 2006 and May 2007.

**Results:**

The 901 PD patients had age range 30 to 88, and median disease duration 50 months. Most (737, 81.8%) used L-dopa (median 375 mg/day), and often added low doses of other antiparkinsonian agents. Among L-dopa-treated patients, the prevalence of motor complications was low (dyskinesias: 8.5%; motor fluctuations: 18.6%), even among patients with disease duration ≥11 years (dyskinesias: 18.1%; motor fluctuations: 42.2%). Higher L-dopa use was associated with higher occurrence of dyskinesias (OR 2.44; 95% CI 1.20-5.13) and motor fluctuations (OR 2.48; 95% CI 1.49-4.14). Initiating PD treatment with L-dopa alone (OR 0.46; 95% CI 0.22-0.95) or in combination with other medications (OR 0.41; 95% CI 0.19-0.87) was associated with less dyskinesia than treatment initiated with non-L-dopa medication.

**Conclusions:**

Many Chinese PD patients are treated with low-dose L-dopa and added low-dose antiparkinsonian agents, with a low prevalence of motor complications, which might be influenced by Chinese culture.

## Background

Parkinson disease (PD) was described in China more than 2400 years ago under the name “wind shaking syndrome,” and traditional medications such as the “Jinya wine” and the “tremor pill” were recommended as early as the Tang (581-682 AD) and Ming (1549-1613 AD) dynasties [1]. Chinese culture covers not only material as medications, but also intangible, i.e. traditional ideas, values, and code of conduct. Culture affects generation after generation, including doctor and decision maker. Traditional features of Chinese culture permeate the care of the elderly afflicted by chronic neurodegenerative diseases. However, most clinical studies on PD treatment have been conducted in Western countries [2-4]. We therefore conducted a large nationwide survey to characterize the clinical features and treatment of PD patients in China and to compare our results with data from Western countries. Patients were recruited from randomly selected hospitals in seven provincial capital cities representing four economic regions of China with an estimated population of 73.2 million.

## Methods

### Design and sampling procedure

In China, as in the West, most neurology and movement disorder clinics are located in university- affiliated hospitals in large cities. Most patients are either already diagnosed or are referred for diagnostic confirmation. We therefore decided to recruit patients from university-affiliated hospitals in provincial capitals. This also ensured the quality of PD diagnosis and clinical data collection. We selected seven provincial capitals from four regions in China with various levels of economic and health care development (average 2010 GDP/capita in Chinese Yuan/medical personnel per 1000 persons in 2009): the Gulf of Bohai (Beijing: 70,234/12.9), Changjiang Delta (Shanghai and Hangzhou: 53,573/9.5 and 5.7), Pearl-River Delta (Guangzhou: 39,978/ 5.0), and Mid-West area (Xian, Wuhan and Jinan: 29,997/4.5, 4.0 and 4.4) [5].

In these regions, there are a total of 349 university-affiliated teaching hospitals and 42 were randomly selected for the study. The number of participants in each city was estimated a priori, in proportion to its population size. The study team included seven field supervisors, one in each city, and 135 physicians (64 senior neurologists, 46 junior neurologists, and 25 graduate students). PD was diagnosed according to the UK Queen Square Brain Bank diagnostic clinical criteria. Between December 2006 and May 2007, we consecutively recruited 907 individuals with either diagnosed (n = 844) or suspected PD (n = 63). The principal investigator (ZZX) reviewed all medical records. Doubtful diagnoses were followed up to one year with additional neurological examinations, and further assessment of the long-term response to L-dopa/DCI, smell test, or brain imaging when necessary. Finally, 901 PD patients were eligible and 6 were excluded due to a final diagnosis of atypical Parkinsonism. All eligible patients agreed to participate and gave written consent. Initial diagnosis and follow-up care was performed at the same participating hospitals in 507 (56.3%) patients. The other 209 patients were referred from community or district hospitals, with an incorrect initial diagnosis in 66.5% (139/209). The Ethics Committee on Human Research at the Peking Union Medical College Hospital approved the study.

### Data collection

Clinical data for 901 consecutive PD patients were collected by 142 interviewers in the 42 participating outpatient clinics following a standard protocol. Data collection included basic demographics, family and medical history, past and current treatment, and comorbidities. We also evaluated motor symptoms and signs using standardized instruments such as Hoehn and Yahr Stage (HYS), the Unified Parkinson’s Disease Rating Scale I-IV (UPDRS), Mini-Mental State Examination (MMSE), Hamilton Depression Scale, and pertinent laboratory tests. All interviewers received a two-day training resulting with videotaped interviews in an inter-rater reliability for UPDRS and HYS of more than 0.90.

### Data analyses

All statistical analyses were performed using SAS (version 8.0) with two-sided alpha of 0.05. In descriptive analyses, we used mean and standard deviation (SD) for normally distributed continuous variables, median and interquartile range (IQ) for continuous variables with skewed distribution, and proportions for categorical variables. We compared demographic and clinical features by disease duration and economic regions. Cardinal variables were compared with the Kruskal-Wallis test, dichotomous variables with 2 × 4 Chi-square test. We used multivariate logistic regression to test the relationship between motor complications and demographic and clinical features. Odds ratios (ORs) and 95% confidence intervals (CIs) were reported accordingly.

## Results

### Clinical features and medication uses

Table 1 shows the demographic and clinical characteristics of 901 Chinese PD patients overall by economic regions. Median age at onset was 61 years. The majority of study participants (80.8%) were at HYS-2 or higher, and median disease duration was 50 months for all patients and 70 months for patients with HYS ≥2.5.

**Table 1:** Demographic and clinical characteristics of 901 PD patients

**Characteristics**	**Total (n** = **901)**		**Gulf of Bohai (n** = **283)**		**Changjian Delta (n** = **275)**		**Pearl**-**River Delta (n** = **110)**		**Mid**-**west Area (n** = **233)**		***P *** **Value**
Men, n (%)	562	(62.4)	180	(63.6)	174	(63.3)	67	(60.9)	141	(60.5)	0.87
Age at survey (yr)	67.0	(15.0)	68.0	(15.0)	66.0	(15.0)	67.5	(13.0)	67.0	(15.0)	0.43
<60, n (%)	254	(28.2)	76	(26.9)	87	(31.6)	27	(24.5)	97	(41.6)	
60-69, n (%)	266	(29.5)	81	(28.6)	111	(40.4)	36	(32.7)	72	(30.9)	
≥70, n (%)	381	(42.3)	126	(44.5)	77	(28.0)	47	(42.7)	64	(27.5)	
Education (yr)	10.7	(4.6)	12.2	(4.2)	10.3	(4.5)	9.4	(5.1)	10.0	(4.3)	<0.001
Occupation, n (%)											<0.001
Labor/ Service, n (%)	392	(43.5)	89	(31.5)	146	(53.1)	58	(52.7)	99	(42.5)	
Administrative, n (%)	253	(28.1)	107	(37.8)	51	(18.5)	24	(21.8)	71	(30.5)	
Professional, n (%)	256	(28.4)	87	(30.7)	78	(28.4)	28	(25.5)	63	(27.0)	
Living on personal income, n (%)	722	(80.1)	261	(92.2)	212	(77.1)	69	(62.7)	180	(77.3)	<0.001
Age of 1^st^ symptom onset (yr)	61.0	(15.0)	60.0	(15.0)	60.0	(16.0)	61.5	(18.0)	63 · 0	(14.0)	0.35
Age at diagnosis (yr)	62.8	(15.6)	61.9	(16.0)	61.7	(15.6)	63.1	(17.8)	63.8	(13.5)	0.29
Months from onset to 1^st^ visit	3.0	(12.0)	4.0	(12.0)	3.0	(9.0)	3.0	(12.0)	3.0	(12.0)	0.11
Tremor as 1^st^ symptom, n (%)	586	(65.4)	185	(65.4)	170	(61.8)	75	(68.2)	156	(67.0)	0.55
Resting tremor, n (%)	758	(84.1)	241	(85.2)	233	(84.7)	88	(80.0)	143	(84.1)	0.64
Rigidity, n (%)	832	(92.3)	270	(95.4)	253	(92.0)	102	(92.7)	207	(88.8)	0.05
Bradykinesia, n (%)	881	(97.8)	281	(99.3)	269	(97.8)	108	(98.2)	223	(95.7)	0.05
Hoehn-Yahr stage, n (%)											<0.001
1.0-1.5	173	(19.2)	44	(15.6)	71	(18.9)	14	(12.7)	44	(18.9)	
2.0-2.5	560	(62.2)	189	(66.8)	151	(58.4)	84	(76.4)	136	(58.4)	
3.0	114	(12.6)	34	(12.0)	45	(12.9)	5	(4.6)	30	(12.9)	
4.0-5.0	54	(6.0)	16	(5.7)	8	(9.9)	7	(6.4)	23	(9.9)	
UPDRS-II, scores	11.0	(8.0)	10.0	(8.0)	10.0	(11.0)	11.0	(7.0)	12.0	(11.0)	<0.001
UPDRS-III, scores	25.0	(20.0)	27.0	(20.0)	23.0	(18.0)	24.0	(19.0)	26.0	(23.0)	0.02
PD duration, months	50.0	(58.0)	56.0	(61.0)	50.0	(56.0)	49.0	(56.0)	44.0	(53.0)	<0.001
Hoehn-Yahr stage 1.0-2.0	37.0	(43.0)	41.0	(48.0)	40.0	(38.0)	37.0	(48.0)	27.0	(45.0)	
Hoehn-Yahr stage 2.5-5	70.0	(70.0)	82.0	(78.5)	72.0	(69.0)	76.0	(75.5)	56.0	(53.5)	
MMSE score < cutoff, n (%)	225	(25.0)	55	(19.4)	61	(22.2)	30	(27.3)	79	(33.9)	0.005
Depressive symptom, n (%)	406	(45.1)	100	(35.3)	113	(41.1)	60	(54.5)	133	(57.1)	0.004

*Abbreviations*: IQ, interquartile range; Values are given as Median (IQ) unless stated otherwise; MMSE cutoff score for aged 55 and over: ≤19 for illiterate, ≤22 for 1-6 years of education, ≤ 26 for ≥7 years of education. Depressive symptom: Hamilton Depression Scale (17 items) ≥9. Cardinal variables were compared with the Kruskal-Wallis test, dichotomous variables with 2x4 –Chi-square test.

**Figure 1:**
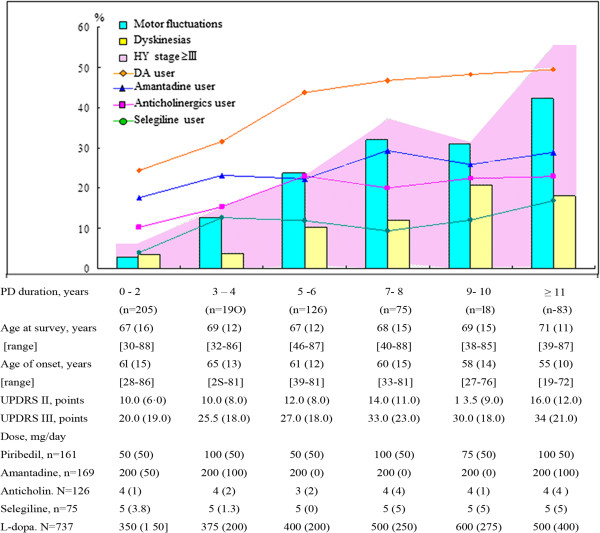
**Prevalence of Motor Complications and Medication uses by PD Duration among L**-**dopa**/**DCI Users.** Abbreviations: Anticholin, Anticholinergics; IQ, Interquartile range; DA, dopamine agonists; UPDRS, Unified Parkinson’s Disease Rating Scale; Values are given as median (IQ) unless stated otherwise.

At enrollment, L-dopa/DCI was the most commonly used antiparkinsonian drug (737, 81.8%), followed by dopamine agonists (296, 32.9%) and amantadine (222, 24.6%) (Table 2). Among L-dopa/DCI users, 596 (80.9%) initiated treatment with L-dopa/DCI and at the time of the survey, 498 patients (67.6%) combined L-dopa/DCI with other antiparkinsonian drugs: dopamine agonists (269, 36.5%), amantadine (169, 22.9%), anticholinergics (126, 17.1%), or selegiline (75, 10.2%) (Figure 1); including former users of L-dopa/DCI plus these drugs, the percentages increased to 46.1%, 41.5%, 39.6%, and 17.2% respectively. Notably, all these drugs were taken at doses lower than those at which they were licensed or recommended for use in published trials from Europe and North America [6]. In our patients, the daily median dose was 375 mg for L-dopa, 200 mg for amantadine, and 5 mg for selegiline, consistently across all regions (Table 2). For dopamine agonists, the median daily doses were 75 mg for piribedil, 0.38 mg for pergolide, 3.75 mg for bromocriptine, and 0.5 mg for pramipexole. At the time of our survey, 32.4% of the 737 L-dopa/DCI users were receiving single-drug therapy, while 46.1% were taking two drugs, 17.8% three drugs, and 3.7% four or five drugs. The median (IQ) dosage of calculated L-dopa/DCI equivalent daily dose (LED) [7] was 450 (350) mg/day and differed between regions. LED increased with disease duration. Chinese herbs were used only in 25 mild patients (UPDRS III = 19.1), in 12 as single-drug therapy and in13 as supplementary therapy.

**Table 2:** Medication use of 901 PD patients

**Medication use**	**Total**		**Gulf of Bohai**		**Changjian Delta**		**Pearl**-**River Delta**		**Mid**-**westArea**		***P *** **Value**
	**(n** = **901)**		**(n** = **283)**		**(n** = **275)**		**(n** = **110)**		**(n** = **233)**		
LED ^a^	450	(350)	500	(330)	435	(360)	525	(353)	375	(330)	0.01
LD/DCI treatment, n (%)	737	(81.8)	227	(80.2)	231	(84.0)	100	(90.9)	179	(76.8)	0.01
LD Dose, mg/d	375	(265)	375	(200)	375	(300)	375	(300)	375	(250)	0.81
LD/DCI Duration, months ^b^	29.5	(51.0)	36.0	(54.0)	34.0	(51.0)	21.0	(45.0)	16.0	(38.0)	<0.001
Dopamine agonists, n (%)	296	(32.7)	98	(34.6)	83	(30.2)	53	(48.2)	62	(26.6)	<0.001
Duration, months ^b^	12.0	(28.0)	18.0	(32.0)	9.5	(23.0)	12.0	(21.0)	8.0	(19.5)	0.004
Amantadine, n (%)	222	(24.6)	88	(31.0)	77	(28.0)	25	(22.7)	32	(13.7)	<0.001
Dose, mg/d	200	(0)	200	(0)	200	(100)	200	(0)	200	(100)	0.07
Duration, months ^b^	21.0	(43.0)	29.5	(62.5)	11.0	(34.0)	10.5	(21.5)	18.5	(53.5)	0.002
Anticholinergics, n (%)	169	(18.8)	43	(15.2)	64	(23.3)	15	(13.6)	47	(20.2)	0.04
Dose, mg/d	4.0	(2.0)	4.0	(3.0)	3.0	(2.0)	4.0	(4.0)	4.0	(4.0)	0.02
Duration, months ^b^	23.0	(51.0)	38.0	(62.0)	25.0	(57.0)	17.0	(21.0)	20.2	(41.0)	0.23
Entacapone, n (%)	55	(6.1)	17	(6.0)	8	(2.9)	11	(10.0)	19	(8.2)	0.02
Dose, mg/d	300	(200)	300	(200)	250	(200)	300	(200)	300	(100)	0.68
Duration, months ^b^	5.5	(14.0)	9.0	(14.0)	9.0	(20.0)	4.0	(5.0)	1.5	(6.5)	0.04
Selegiline, n (%)	87	(9.7)	36	(12.7)	36	(13.1)	13	(11.8)	2	(0.9)	<0.001
Dose, mg/d	5.0	(2.5)	5.0	(2.5)	5.0	(5.0)	5.0	(0.0)	5.0	(0.0)	0.63
Duration, months ^b^	15.0	(27.0)	24.0	(23.0)	12.0	(33.0)	7.5	(22.0)	15.0	(0.0)	0.87
All LD/DCI users (n=737)											
Motor fluctuation, n (%)	137	(18.6)	47	(20.7)	50	(21.6)	11	(11.0)	29	(16.2)	0.09
Dyskinesias, n (%)	63	(8.6)	22	(9.7)	15	(6.5)	11	(11.0)	15	(8.4)	0.49

*Abbreviations*: LED: L-dopa/DCI equivalent daily dose; LD: L-dopa; IQ: Interquartile range.

Values are given as Median (IQ) unless stated otherwise;

^a^ n = 806.

^b^ Data are available for duration of medication user: n = 726 for L-dopa, n = 267 for dopaming agonists, n = 211 for amantadine, n = 159 for anticholinergics, n = 46 for entacapone, and n = 65 for selegiline.

The four economic regions differed in demographic and PD-related characteristics (Table 1). Compared to patients from other regions, those from Gulf of Bohai were more likely to be professionals or administrators with a higher education level and financial independence. However, they did not differ in age of onset or diagnosis, or main PD symptoms or signs. Mid-West was the least developed region. Compared to this region, PD patients from the Gulf of Bohai had longer disease duration and yet better HYS and activities of daily living (UPDRS-II) scores. This regional difference of disease duration was evident even in stratified analyses by HYS subgroups. The use of amantadine and selegiline was also related to economic development (Table 2).

### Disease progression and motor complications

Of the 737 L-dopa/DCI users, 137 (18.6%) had motor fluctuations and 63 (8.6%) had dyskinesias. The prevalence of motor complications increased with disease duration (Figure 1): the prevalence of dyskinesia was 3.4% among patients with 3 years of disease, 6.2% among patients with 4-6 years of disease as compared to 18.1% among patients who had had the disease for ≥11 years; the corresponding numbers for motor fluctuations were 2.9%, 14.9%, and 42.2%, respectively. Among young-onset patients (<50 years, n = 111), median disease duration was 6.4 years, HYS 2, and the prevalence of dyskinesias and motor fluctuations 12.6% and 22.5%, respectively. Of the 137 patients with motor fluctuations, 38 had dyskinesias. As a proxy for the severity of motor fluctuations, the median score of UPDRS item 39 (off hours in total waking time) was 1 for both groups of patients.

Although the overall dosages of L-dopa/DCI and total LED were low, they increased with disease duration, from 350 mg/day or 375 mg/day for patients with <3 years of the disease to 500 mg/day or 670 mg/day for those with ≥11 years. The dosage of other antiparkinsonian medications remained steadily low throughout the course of the disease, but the proportion of adjuvant medication use increased with disease duration (Figure 1). At enrollment, the proportion of adjuvant therapy in addition to L-dopa/DCI among patients with <3 years of the disease was 24.4% for dopamine agonists, 17.6% for amantadine, 3.9% for selegiline, and 10.2% for anticholinergics. In contrast, the proportions among patients with ≥11 years of disease duration was 49.4% for dopamine agonists, 28.9% for amantadine, 16 · 9% for selegiline, and 22.9% for anticholinergics.

In multivariate analyses, HYS ≥3 and L-dopa doses ≥400 mg/d were associated with higher occurrence of dyskinesia (Table 3), whereas initiation of L-dopa/DCI alone or added to other medication was associated with lower occurrence of dyskinesia. PD duration ≥5 years, HYS ≥3, and L-dopa doses ≥600 mg/d were each associated with higher occurrence of motor fluctuations. On the other hand, initiation of treatment with L-dopa/DCI and delayed initiation of treatment one year after onset were not associated with higher occurrence of motor fluctuations.

**Table 3:** **Multivariate regression analyses of motor complications correlates among 737 L**-**dopa**/**DCI users**

**Variable**		**Dyskinesias**		***P *** **Value**	**Motor fluctuations**		***P *** **Value**
		**Yes** / **no**	**OR (95% CI)**		**Yes** / **no**	**OR (95% CI)**	
Age at onset (yr)	≥59	30 / 420	Reference	0.15	68 / 382	Reference	0.13
	<45	11 / 49	2.32 (1.00 – 5.41)		14 / 46	1.20 (0.57 – 2.53)	
	45-58	22 / 205	1.31 (0.70 – 2.46)		55 / 172	1.63 (1·02 – 2·61)	
PD duration (yr)	<5	15 / 399	Reference	0.36	30 / 384	Reference	0.02
	5-9	28 / 188	1.88 (0.79 – 4.51)		64 / 152	2.52 (1.31 – 4.85)	
	≥10	20 / 87	1.82 (0.59 – 5.59)		43 / 64	2.46 (1.05 – 5.76)	
Hoehn -Yahr stage	<3	38 / 540	Reference	0.03	76 / 502	reference	<0.001
	≥3	35 / 134	2.03 (1.08 – 3.82)		61 / 98	2.58 (1.62 – 4.11)	
LD dose, mg/d	<400	16 / 370	Reference	0.02	39 / 347	Reference	0.002
	400-599	21 / 149	2.48 (1.20 – 5.13)		37 / 133	1.69 (0.99 – 2.91)	
	≥600	26 / 155	2.44 (1.20 – 4.98)		61 / 120	2.48 (1.49 – 4.14)	
LD/DCI duration (yr)	<2	14 / 294	Reference	0.44	23 / 285	Reference	0.07
	2-4	15 / 215	1.04 (0.47 – 2.34)		36 / 194	1.58 (0.86 – 2.87)	
	≥5	34 / 165	1.73 (0.66 – 4.51)		78 / 121	2.42 (1.14 – 5.12)	
Months to initiate	≤12	27 / 339	Reference	0.87	66 / 300	Reference	0.66
LD/DCI use	>12	36 / 439	0.95 (0.49 – 1.83)		71 / 300	0.89 (0.54 – 1.48)	
Initial treatment	non LD/DCI	22 / 119	Reference	0.04	35 / 106	Reference	0.42
	LD/DCI only	22 / 259	0.46 (0.22 – 0.95)		53 / 228	0.76 (0.42 – 1.36)	
	LD/DCI + others	19 / 296	0.41 (0.19 – 0.87)		49 / 266	0.67 (0.37 – 1.22)	

*Abbreviations*: LD, L-dopa; DCI, decarboxylase Inhibitor; OR, odds ratios; CI, confidence interval.

Model adjusted for gender, age at onset, PD duration, Hoehn -Yahr stage, levodoap dose, LD/DCI duration, region, months to initiate LD/DCI use, and initial treatment.

## Discussion

To the best of our knowledge, this is to date the largest hospital-based clinical survey of PD patients in China. We consider our ambulatory PD population as representative of urban regions in China since sample size was estimated a priori in proportion to regional population size; nearly all patients with neurological symptoms are managed primarily by university outpatient clinics or will be referred to them for final diagnosis and treatment. We may have missed patients with very advanced PD while those with early disease may have been somewhat overrepresented as a consequence of the improvements in the Chinese health assurance and social security system. Comparisons with Western countries therefore need to be made within classes of illness duration. Like their counterparts in Western countries, PD patients in China are predominantly men with average symptom onset in the early sixties; bradykinesia and rigidity are the main signs and most patients are treated with L-dopa/DCI, often in combination with other antiparkinsonian agents [8-13]. Unlike PD patients in Western countries, many Chinese patients take medications at lower dosages and have a lower prevalence of medication-related motor complications.

China is a big country and economic development varies greatly across the country. Our patients were recruited from 42 university-affiliated teaching hospitals randomly selected from all teaching hospitals in seven capital cities in four areas with various levels of economic development. We identified some regional differences in population and clinical characteristics of PD patients that correlated with economic development. Compared to the Mid-West, the least economically developed region, patients in the well-developed Gulf of Bohai were less likely to have HYS ≥ 3 and had lower UPDRS-II scores, despite a much longer mean disease duration. Patients in the Gulf of Bohai were also more likely to be treated with amantadine and selegiline in addition to L-dopa/DCI.

Studies in Western countries have reported that approximately 50-90% of PD patients develop motor complications after 5-10 years of dopaminergic replacement therapy [2-4,14-20]. We, however, observed a much lower prevalence of motor complications among Chinese PD patients, overall 8.6% for dyskinesias and 18.6% for motor fluctuations. The difference in dyskinesias remained even when comparing patients with similar disease duration (median 4-6 years): 6.2% in Chinese patients compared to 27.8% (10.3-40%) identified by Ahlskog and Muenter in their review article [2] or (median 14 years) 18.1% of dyskinesias in Chinese patients compared to 58% in UK patients [20]. The difference in motor fluctuations was less dramatic, but still 42.2% compared to 50% [20]. A similar difference was found when we stratified by age at onset: for example, only 12.6% of our early-onset cases (<50 years) had dyskinesias compared to 40% in USA [21]. It is well established that these motor complications are the result of L-dopa treatment [3,4]. A notable difference between Chinese patients and those in the West is that Chinese patients took L-dopa (as well as other medications) at low dosages across all regions. Dosages also remained low through the course of the disease. Further, in our study, the presence of dyskinesias was related to the dose of L-dopa, a finding consistent with that of other studies [3,4]. Therefore, it is reasonable to suspect that this low prevalence of motor complications may be in part due to low-dose L-dopa/DCI treatment (median 400 mg/d).

In addition to L-dopa/DCI, 67.6% of Chinese patients also used other antiparkinsonian agents as adjunctive therapy at the time of our survey, also at low doses. As disease duration increased, patients used more classes of these agents, but still at low doses. Dopamine agonists and amantadine were the most commonly used adjunctive medications. Previous studies showed that amantadine might delay the need for L-dopa/DCI in early PD and provide an antidyskinetic effect in advanced patients [21-24]. In our study, 22.9% of Chinese PD patients currently used, and 41.5% had ever used, amantadine combined with L-dopa, a proportion much higher than in Western patients (e.g., current users in the UK 5%, in Italy 11.4%, and in Spain 18.8%) [15,16,25]. Adjunctive selegiline can reduce the need for high-dose L-dopa/DCI and may thus decrease the risk for motor fluctuations, although at higher dosages (10 mg/d) it may induce dyskinesias [4,26,27]. In Europe, the reported proportion of adjunctive selegiline use ranges from 8.1 to 34.4% [15,16,27]. In the present study, 10.2% of patients currently used, and 17.2% had ever used, selegiline, and typically at low doses (median 5 mg/d). Finally, a recent meta-analysis confirmed that adjuvant therapy with dopamine agonists reduced the need for higher L-dopa/DCI dose, although it increased dyskinesias in patients with later-stage PD [7]. Dopamine agonists were the most commonly used adjunctive drug among Chinese patients, but again at low doses consistently throughout disease duration. For example, the median dose of piribedil remained at 50-100 mg/day, that of amantadine at 200 mg/day, and that of selegiline at 5 mg/day.

The choice of multiple but low-dose medication may reflect Chinese philosophy regarding the treatment of chronic disease: “a small stream runs far”. Chinese treatment guideline of PD recommended “never go for full effective dose” [28,29]. Many Chinese patients start L-dopa/DCI in the low therapeutic range. The higher dose that is standard in Western countries may achieve satisfactory motor control sooner, but this may be at the expense of motor complications.

Deferral of L-dopa/DCI treatment has been recommended in order to delay the onset of motor complications in patients younger than 70 years [22]. However, in the current study, motor complications were less frequent in patients who started with L-dopa/DCI alone or in combination with other drugs than in those who started with other drugs. L-dopa/DCI deferral was not associated with a lower occurrence of dyskinesias or motor fluctuations. Similar clinical observations have been made in other studies [3,4,20,30]. Therefore, early initiation of low-dose L-dopa/DCI may help to reduce the future occurrence of motor complications. In support of this view, the lifetime cumulative dose of L-dopa use was not related to PD pathology among patients with younger age at onset and longer duration [31]. In summary, early introduction of low-dose symptomatic therapy may preserve basal ganglia compensatory mechanisms [4,31].

A community-based study has recommended using the emergence of axial symptoms (HYS 3) as a surrogate of rapid progression [14]. Compared to studies in the West with similar disease duration, HYS ≥3 disease was not more common in our patients. For example, in patients with a median 14 years disease duration, the average HYS was 3.0 in our study compared with 3.1-3.4 in other studies [20]. in those with about 9 years of disease duration, the average HYS was 2.6 in our population compared to 2.8 in others [11,12]. Median off times in our Chinese patients with motor fluctuations were at a moderate level. Moreover, compared with patients from other regions, those from Bohai had a higher prevalence of antiparkinsonism drug combinations (with a similar low dosage for each form), resulting in higher LED. Patients in Bohai had longer disease duration, especially for more severe subjects (HYS ≥2.5), but their activities of daily living were not worse and their quality of life was possibly better, moreover cognitive impairment and depressive symptoms were less frequent than in patients from other areas. Therefore, the low prevalence of motor complications among Chinese patients was not at the expense of poor quality of life, rapid motor deterioration, or prolonged off-times. The Chinese treatment strategy adequately controlled motor signs and helped maintain quality of life.

A multitude of neurotransmitters and receptors, including glutamatergic, opioid, serotonergic, g-aminobutyric acid (GABA)-ergic, adenosine, cannabinoid, adrenergic, histaminergic, and cholinergic systems, have been reported to play a role in the etiology of dyskinesias [32]. The traditional approaches, which usually focus on a single target, may not be appropriate, although non-physiologic, pulsatile dopamine release seem to play critical roles. Our clinical observation showed that combined antiparkinsonian drugs with different mechanisms of action at a small but effective dose reduced the risk of dyskinesias. It might be explained that the combined treatment targets simultaneously more than one neurotransmitter system, through transcription factors and intracellular signaling to alter expression of dyskinesia [32].

Although this is the largest clinical survey of PD patients in China, our study has several limitations. It is a cross-sectional analysis of patients with various disease durations. Therefore we were unable to make a direct causal inference that low-dose medication leads to fewer motor complications. Although our study is a good reflection of patients from urban areas, we might have missed some patients from rural areas or small cities. However, as explained previously, patients in rural areas of small cities often lack medical resources and have to seek help in large cities for PD diagnosis and treatment. Finally, in China there are no family doctors; family-based home care is the traditional customary, but not institutional care for the elderly. Long-term healthcare is often provided by family members, while patients with long-term PD may see a neurologist less often, with the result that our study may have preferentially included less patients with longer disease duration. However, 42.2% of our patients were ≥70 years old and 41.0% had average disease duration of 9.2 (5-27) years.

## Conclusion

In summary, this large clinical survey provides important data on PD clinical practice in China. The difference observed in medication use and also in the prevalence of motor complications between patients in China and patients in the West may reflect cultural differences in medical treatment. The possibility that low dose L-dopa/DCI use in conjunction with other low-dose antiparkinsonian medications may lead to fewer motor complications should be further investigated in future prospective studies.

## Competing interests

Dr. ZX Zhang, a neurologist at PUMC Hospital, reports no conflicts of interest. Dr. H Chen from the US NIH reports no conflicts of interest. Drs. SD Chen, SG Sun, M Shao, QM Qiu, BR Zhang, and YM Liu, sub-PI from 6 provincial cities, report no disclosures. Drs. X Wan, L Li, X Chen, HB Wen, and Q Xu work at PUMC, and HB Chen in Beijing, and ZG Liu, J Wang and G Wang in Shanghai report no disclosures.

## Authors’ contributors

Dr. ZXZ: drafting/revising the manuscript, study concept and design, analysis and interpretation of data, acquisition of data, acquisition of funding, and study supervision. Dr. HC: revising the manuscript, study concept and design, interpretation of data, and acquisition of funding. Dr. XW and QX: interpretation of data and data statistical analysis. Drs. SDC, MS, SGS, QMQ, BRZ, YML, LL, HBW, XC, HBC, ZGL, JW, and GW collected the data. All authors contributed to the design of the study, and interpretation of data. All authors read and approved the final manuscript.
